# Comparative Effects of Coenzyme Q10 or *n*-3 Polyunsaturated Fatty Acid Supplementation on Retinal Angiogenesis in a Rat Model of Oxygen-Induced Retinopathy

**DOI:** 10.3390/antiox7110160

**Published:** 2018-11-09

**Authors:** Kay D. Beharry, Charles L. Cai, Faisal Siddiqui, Sara Chowdhury, Christina D’Agrosa, Gloria B. Valencia, Jacob V. Aranda

**Affiliations:** 1 Department of Pediatrics, Division of Neonatal-Perinatal Medicine, State University of New York, Downstate Medical Center, Brooklyn, NY 11203, USA; charles.cai@downstate.edu (C.L.C.); faisal.siddiqui@downstate.edu (F.S.); sara.chowdhury@downstate.edu (S.C.); christina.d’agrosa@downstate.edu (C.D.); gloria.valencia@downstate.edu (G.B.V.); jacob.aranda@downstate.edu (J.V.A.); 2 Department of Ophthalmology; State University of New York, Downstate Medical Center, Brooklyn, NY 11203, USA; 3 State University of New York Department of Ophthalmology Eye Institute, New York, NY 10062, USA

**Keywords:** coenzyme Q10, insulin-like growth factor-I, *n*-3 polyunsaturated fatty acids, neonatal intermittent hypoxia, oxygen-induced retinopathy, vascular endothelial growth factor

## Abstract

Neonatal intermittent hypoxia (IH) or apnea afflicts 70% to 90% of all preterm infants <28 weeks gestation, and is associated with severe retinopathy of prematurity (ROP). We tested the hypotheses that coenzyme Q10 (CoQ10) or omega-3 polyunsaturated fatty acids (*n*-3 PUFAs) supplementation during neonatal IH reduces the severity of oxygen-induced retinopathy (OIR). Newborn rats were exposed to two IH paradigms: (1) 50% O_2_ with brief hypoxia (12% O_2_); or (2) 21% O_2_ with brief hypoxia, until postnatal day 14 (P14), during which they received daily oral CoQ10 in olive oil, *n*-3 PUFAs in fish oil, or olive oil only and compared to room air (RA) treated groups. Pups were examined at P14, or placed in RA until P21. Retinal angiogenesis, histopathology, and morphometry were determined. Both IH paradigms produced severe OIR, but these were worsened with 50/12% O_2_ IH. CoQ10 and *n*-3 PUFAs reduced the severity of OIR, as well as ocular growth factors in both IH paradigms, but CoQ10 was more effective in 50/12% O_2_ IH. Supplementation with either CoQ10 or *n*-3 PUFAs targeting IH-induced retinal injury is individually effective for ameliorating specific characteristics consistent with ROP. Given the complexity of ROP, further studies are needed to determine whether combined CoQ10 and *n*-3 PUFAs supplementation would optimize their efficacy and result in a better outcome.

## 1. Introduction

Retinopathy of prematurity (ROP) is a leading cause of childhood blindness worldwide [1]. Late complications of ROP include the development of other ocular diseases such as glaucoma, amblyopia, strabismus, myopia, and retinal detachment [2,3], resulting in financial and emotional burdens [4,5,6,7]. ROP is a developmental vascular disorder characterized by the abnormal growth of retinal blood vessels in extremely low gestational age neonates (ELGANs) who are ≤28 weeks gestation [8,9,10,11]. While the exact etiology of ROP is unknown, excessive and high levels of oxygen, oxidative distress, inflammation, poor nutrition, dysregulated growth factors, and neonatal intermittent hypoxia (IH), contribute to its severity.

Neonatal IH is defined as brief, recurrent arterial oxygen desaturations [12,13,14,15,16], and is one of the major factors associated with severe ROP in ELGANs requiring oxygen therapy [17,18,19,20,21,22,23]. An IH event is usually defined as a decline in SaO_2_ by 5% lasting <3 min in duration [13,14,15]. Reoxygenation following an IH event can occur in normoxia or hyperoxia, but whether the effects of IH with resolution in normoxia is less deleterious than those in hyperoxia, remains to be determined. In the United States, the estimated incidence of any ROP is 68% in preterm infants weighing less than 1251 g [4]. These infants experience the highest incidence of IH, and are more vulnerable to poor postnatal growth [24]. ELGANs often develop deficits in *n*-3 polyunsatuarated fatty acids (PUFAs) such as docosahexaenoic acid (DHA) [25,26,27], which is thought to contribute to major neonatal diseases, including ROP. Dietary supplementation with the *n*-3 PUFAs appears to be protective, and reduces the severity of neonatal diseases [28,29], presumably by improving growth and reducing oxidative distress [30]. 

ELGANs are highly susceptible to oxidative distress and aggression due to oxygen therapy, mechanical ventilation, immature antioxidant systems, and reduced placental transfer of antioxidants, including coenzyme Q10 (CoQ10). CoQ10 is a powerful antioxidant that directly scavenges oxygen free radicals or indirectly regenerates vitamin E. In the mitochondria, CoQ10 is involved in oxidative phosphorylation (OXPHOS) and energy production [31,32]. CoQ10 is found in tissues with high energy demands, including the eyes [33,34]. It has bioenergetic properties, is involved in energy production, and is involved in the prevention of membrane phospholipid peroxidation and free radical oxidation [35]. Numerous studies have shown the benefits of CoQ10, including reversing mitochondrial dysfunction and increasing cellular metabolism [36,37,38,39]. Despite reports of the beneficial effects of CoQ10 and *n*-3 PUFAs supplementation on oxidative distress and retinal health, no previous studies have examined or compared their possible beneficial efficacy for improving the severity of oxygen-induced retinopathy (OIR) resulting from neonatal IH. To this end, we conducted a series of experiments to test the hypothesis that CoQ10 or *n*-3 PUFAs supplementation can reduce the severity of IH-induced OIR. We employed two different, but clinically relevant, IH paradigms to induce severe OIR and examine the protective effects of CoQ10 or *n*-3 PUFAs supplementation on retinal angiogenesis using a number of quantitative analyses. 

## 2. Materials and Methods 

### 2.1. Animals

All of the experiments were approved by the State University of New York, Downstate Medical Center Institutional Animal Care and Use Committee, Brooklyn, NY (Protocol #17-10517). Animals were cared for according to the guidelines of the United States Department of Agriculture and the Guide for the Care and Use of Laboratory Animals. Certified infection-free, timed-pregnant Sprague Dawley rats were purchased from Charles River Laboratories (Wilmington, MA, USA) at 18 days gestation. The animals were housed in an animal facility with a 12-h day/12-h night cycle and provided a standard laboratory diet and water ad libitum until delivery of their pups. All of the procedures were performed in accordance with the Association for Research in Vision and Ophthalmology statement on the Use of Animals in Ophthalmic and Vision Research.

### 2.2. Experimental Design

Within 2–4 h of birth, newborn rat pups delivering on the same day were pooled and randomly assigned to expanded litters of 18 pups/litter (nine males and nine females), as previously described [40]. Animals were exposed to neonatal intermittent hypoxia (IH) from postnatal day 0 (P0) to P14, or allowed to recover from IH in room air (RA) until P21. Methods and daily doses of oral CoQ10 and *n*-3 PUFAs were conducted as previously described [40]. Supplementation occurred only from P0–P14. There was no supplementation from P14 to P21. RA littermates were raised in atmospheric oxygen from P0 to P14 or P0 to P21, were similarly supplemented, and served as age-matched controls. The caecal period in rats represent the period from conception to eye opening. This usually occurs at P14, and coincides with the maturation of the retinal neural circuitry. The percentage of rats that opened their eyes at P14 was also recorded.

### 2.3. Neonatal Intermittent Hypoxia (IH) Profiles

Episodes of reoxygenation following an IH event may occur in normoxia or hyperoxia. The two IH paradigms and confirmation of oxygen saturation have been previously described [40,41,42,43,44,45,46]. 

### 2.4. Sample Collection and Processing

Eighteen groups of rats were studied (*n* = 18 pups/group; nine males and nine females; 36 eyes): (1) olive oil in RA (P14); (2) olive oil in RA (P21); (3) CoQ10 in RA (P14); (4) CoQ10 in RA (P21); (5) *n*-3 PUFAs in RA (P14); (6) *n*-3 PUFAs in RA (P21); (7) olive oil in 50/12% O_2_ IH (P14); (8) olive oil in 50/12% O_2_ IH (P21); (9) CoQ10 in 50/12% O_2_ IH (P14); (10) CoQ10 in 50/12% O_2_ IH (P21); (11) *n*-3 PUFAs in 50/12% O_2_ IH (P14); (12) *n*-3 PUFAs in 50/12% O_2_ IH (P21); (13) olive oil in 21/12% O_2_ IH (P14); (14) olive oil in 21/12% O_2_ IH (P21); (15) CoQ10 in 21/12% O_2_ IH (P14); (16) CoQ10 in 21/12% O_2_ IH (P21); (17) *n*-3 PUFAs in 21/12% O_2_ IH (P14); and (18) *n*-3 PUFAs in 21/12% O_2_ IH (P21). At euthanasia, eyes were enucleated and rinsed in ice-cold phosphate-buffered saline (PBS, pH 7.4) on ice. Enucleation was performed with the use of iris forceps and scissors for separation of the eyes from the surrounding connective tissue, nerves, and muscle. For assessment of ocular growth factors, 12 eyes were used (six males and six females). Two eyes were pooled for a total of six vitreous fluid, retinal, and choroidal samples (three males and three females). The vitreous fluid was obtained by gently perforating the eyes and centrifugation at 5000 rpm at 4 °C for 15 min; the vitreous fluid was collected in a collection Eppendorf tube. Dissection and harvesting of the retinal and choroidal tissues were conducted as previously described [44]. For assessment of retinal vascular density, whole eyes (six per group, three males and three females) were placed in 4% paraformaldehyde (PFA) PH 7.4 for 90 min prior to flatmounting and adenosine diphosphatase (ADPase) staining of the superficial vasculature. Images were used for the WimRetina quantification of the retinal vasculature. For assessment of the retinal astrocyte and vascular integrity, whole eyes (six per group, three males and three females) were flatmounted in ice-cold PBS pH 7.4 and stained for glial fibrillary acidic protein (GFAP) and isolectin B4. For retinal histopathology and morphometry, whole eyes (six per group, three males and three females) were placed in 10% phosphate-buffered formalin and sent to Histowiz, Inc. (Brooklyn, NY, USA) for processing, embedding, and H&E staining using standard laboratory techniques. The remaining six eyes (three males and three females were kept frozen at −80 °C in the biorepository for future use.

### 2.5. Assay of Ocular Growth Factors

Vascular endothelial growth factor (VEGF), soluble VEGF receptor-1 (sVEGFR-1), and insulin-like growth factor-1 (IGF-I) levels were determined in the ocular samples (vitreous fluid, retina, and choroid) using commercially available rat VEGF and IGF, and mouse VEGFR-1 Quantikine enzyme-linked immunosorbent assay (ELISA) kits, respectively, purchased from R&D Systems (Minneapolis, MN, USA). All of the samples were processed and assayed according to the manufacturer’s protocol, and the mouse VEGFR-1 ELISA kits detected rat VEGFR-1. Levels in the retinal and choroidal tissue homogenates were standardized using total cellular protein levels. 

### 2.6. Total Cellular Protein Levels

On the day of assays, an aliquot (10 µL) of the retinal and choroid homogenates was utilized for total cellular protein levels using the Bradford method (Bio-Rad, Hercules, CA, USA) with bovine serum albumin as a standard.

### 2.7. Retinal Flatmounts

Eyes were enucleated and placed in 4% PFA (pH 7.4) on ice. The eyes were placed in the refrigerator for 90 min, after which they placed in ice-cold phosphate-buffered saline (PBS) on ice. Following removal of the cornea and lens, the retina was separated from the sclera, cut into four quadrants, and flattened. Retinas dedicated for ADPase staining were immersed in 4% PFA and stored overnight at 4 °C. Retinas dedicated for glial fibrillary acidic protein (GFAP) and isolectin B4 double staining were immersed in PBS/Triton X-100 (TXPBS).

### 2.8. ADPase Staining of the Retinas

After 24 h of incubation in 4% PFA, the retinas were washed in tris-maleate buffer (pH 7.2) on ice prior to incubation in an ADPase incubation medium containing 3.0 nM of lead nitrate and 6.0 mM of magnesium chloride (Sigma Chemical Co., St. Louis, MO, USA) in tris-maleate buffer (pH 7.2). After incubation, the retinas were washed with tris-maleate buffer prior to the addition of diluted ammonium sulfide (Fisher Scientific, Silver Spring, MD, USA) for 1 min. The retinas were washed again in tris-maleate buffer and flatmounted on a microscope slide with PBS and glycerin. All of the images were captured using an Olympus BX53 microscope, DP72 digital camera, and CellSens Dimension imaging software (version 2.1) from Olympus America, Inc. (Center Valley, PA, USA), attached to a HP Z44 computer. 

### 2.9. GFAP and Isolectin B4 Staining

GFAP and isolectin B4 double staining of the retinal flatmounts was conducted as previously described [44]. 

### 2.10. Vascular Density Quantification

Quantification of retinal angiogenesis was carried out in a masked manner using several quantitative methods: (1) WimRetina analysis; (2) tortuosity index; (3) vessel diameter; (4) number of endothelial cells present in the nerve fiber layer (NFL)/ganglion cell layer (GCL); and (5) total retinal thickness, and thickness of the NFL/GCL, inner plexiform layer (IPL), inner nuclear layer (INL), and outer nuclear layer (ONL); and (6) retinal scoring, as previously described [44,47]. 

### 2.11. Retinal Scoring

ADPase-stained retinal images (*n* = 24 images per group) were scored in a masked manner by three neonatology fellows who were trained in the rat retinal scoring system that was modified for our neonatal IH model from the scoring system developed by Higgins et al. [48]. The scoring criteria included vascular tufts, retinal overgrowth (vessels growing beyond the avascular zone and into the ciliary body), retinal hemorrhage, vessel tortuosity, enlarged vessels, and persistence of hyaloid vessels. For scoring, each of the four quadrants of each retina was divided into equally-sized sections to represent a total of 12 “clock hours”. A score of zero was given if none of the characteristics were found in any of the clock hours; a score of one was given if found in <3 clock hours; a score of two was given if found in 3–5 clock hours; a score of three was given if found in 6–8 clock hours; and a score of four was given if found in 9–12 clock hours. The retinopathy score was calculated by the sum of points for each criterion. The maximum score was 24 retinas, and a score of ≥18 was considered severe OIR. 

### 2.12. Statistical Analysis

Differences among the RA and IH groups, and differences among the supplemental groups within each oxygen environment were analyzed using two-way analysis of variance (ANOVA) with Dunnett’s post-hoc tests, following the Bartlett’s test for normality. The Kruskal–Wallis nonparametric test with Dunn’s multiple comparison were used for non-normally distributed data. The percentage of retinas that scored >18 in each group was calculated and analyzed using Fisher’s exact test. Data were analyzed using the Statistical Package for the Social Sciences (SPSS) software, version 16.0 (SPSS Inc., Chicago, IL, USA) and are reported as mean ± standard deviation (SD). A *p*-value of <0.05 was considered as statistically significant.

## 3. Results

### 3.1. Eye-Opening at P14

Eye-opening in rats concurs with maturation of the retinal neural circuitry and usually occurs at or around P14. Table 1 shows that *n*-3 PUFAs significantly induced eye opening at P14 in the RA and 50/12% O_2_ IH groups compared to CoQ10 and olive oil controls. CoQ10 induced eye opening in the 21/12% O_2_ IH-exposed group. 

### 3.2. VEGF Levels

**Figure 1:**
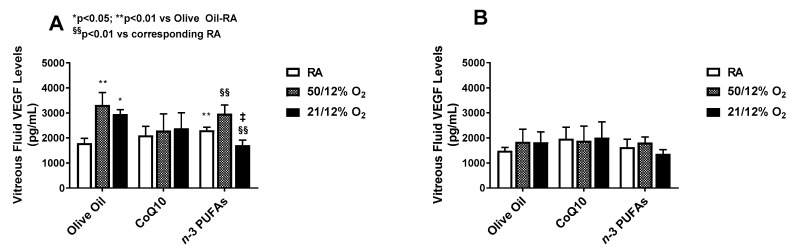
Effect of CoQ10 or *n*-3 PUFA supplementation in IH on vitreous fluid VEGF levels. Data are mean ± SD. *n* = six samples/group (* *p* < 0.05; ** *p* < 0.01 vs. olive oil in RA; ^‡^ *p* < 0.01 vs. olive oil in 21/12% O_2_; and ^§§^ p < 0.01 vs. corresponding treatment in RA). IH (intermittent hypoxia); RA (room air); VEGF (vascular endothelial growth factor).

VEGF is one of the most potent angiogenic growth factors responsible for normal and pathologic retinal angiogenesis. The vitreous fluid is a reservoir for growth factors. VEGF levels in the vitreous fluid at P14 (A) and P21 (B) are presented in Figure 1.

Vitreous fluid VEGF levels were significantly increased in the olive oil groups exposed to both IH paradigms, although exposure to 50/12% O_2_ IH caused the highest increase. Supplementation with CoQ10 and *n*-3 PUFAs in 50/12% O_2_ IH did not significantly reduce VEGF levels in the vitreous fluid compared to olive oil. However, reductions with *n*-3 PUFAs occurred in the 21/12% O_2_ IH group at P14. No differences in vitreous fluid VEGF levels among the groups were noted at P21. 

**Figure 2:**
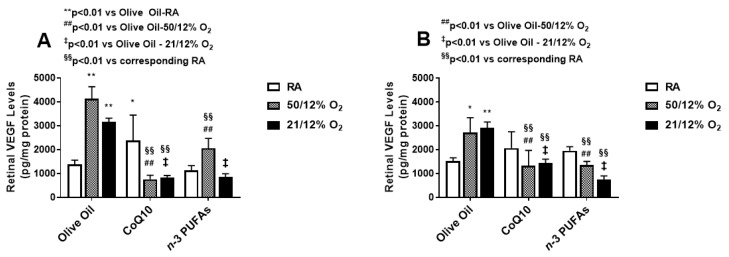
Effect of CoQ10 or *n*-3 PUFA supplementation in IH on retinal VEGF levels. Data are mean ± SD. *n* = six samples/group (* *p* < 0.05; ** *p* < 0.01 vs. olive oil in RA; ^##^ *p* < 0.01 vs. olive oil in 50/12% O_2_; ^‡^ *p* < 0.01 vs. olive oil in 21/12% O_2_; and ^§§^ *p* < 0.01 vs. corresponding treatment in RA). RA (room air); VEGF (vascular endothelial growth factor).

VEGF levels in the retinal homogenates at P14 (A) and P21 (B) are presented in Figure 2. Similar to the vitreous fluid, exposure to both IH paradigms increased retinal VEGF levels at P14, with predominantly higher levels in the 50/12% O_2_ IH group supplemented with olive oil. CoQ10 and *n*-3 PUFAs suppressed retinal VEGF levels, but CoQ10 was more effective in 50/12% O_2_ IH. At P21, the levels of retinal VEGF remained elevated, and the effects of CoQ10 and *n*-3 PUFAs on retinal VEGF levels remained sustained.

**Figure 3:**
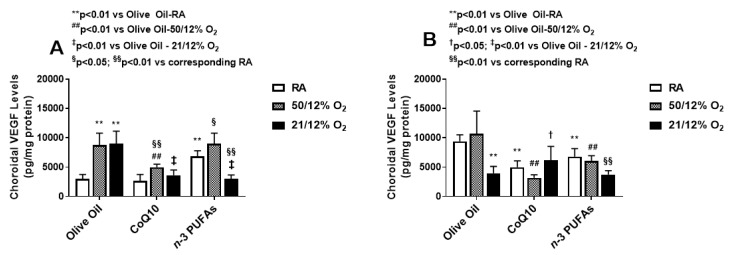
Effect of CoQ10 or *n*-3 PUFA supplementation in IH on choroidal VEGF levels. Data are mean ± SD. *n* = six samples/group (** *p* < 0.01 vs. olive oil in RA; ^##^ *p* < 0.01 vs. olive oil in 50/12% O_2_; ^†^ *p* < 0.05, ^‡^ *p* < 0.01 vs. olive oil in 21/12% O_2_; and ^§^ *p* < 0.05; ^§§^ *p* < 0.01 vs. corresponding treatment in RA). RA (room air); VEGF (vascular endothelial growth factor).

VEGF levels in the choroidal homogenates at P14 (A) and P21 (B) are presented in Figure 3. Levels of VEGF in the choroid increased substantially at P14 in the groups exposed to IH and supplemented with olive oil, and surpassed that of the retina. While CoQ10 suppressed choroidal VEGF levels in both IH paradigms, *n*-3 PUFAs were effective only in the 21/12% O_2_ IH group. Interestingly, *n*-3 PUFA supplementation resulted in higher choroidal VEGF levels in RA and 50/12% O_2_ IH. At P21, the levels declined with both CoQ10 or *n*-3 PUFA supplementation, but remained elevated in the 50/12% O_2_ IH group supplemented with olive oil. Together, these findings may make *n*-3 PUFAs appear to be more effective for the suppression of ocular VEGF overproduction in 21/12% O_2_ IH than 50/12% O_2_ IH, and increase VEGF levels in the choroid. On the contrary, CoQ10 appears to be more effective in suppressing VEGF levels during 50/12% O_2_ IH in both the retina and choroid.

### 3.3. Ocular sVEGFR-1

Soluble VEGFR-1 (sVEGFR-1) is an endogenous negative regulator of VEGF that is a splice variant of membrane type. It acts as a VEGF “trap” by binding VEGF and preventing its availability to the membrane VEGF receptors. sVEGFR-1 levels in the vitreous fluid, retina, and choroid are presented in Table 2. Similar to the VEGF response at P14, sVEGFR-1 levels were higher in the ocular compartment of the control groups exposed to both IH paradigms, and were significantly reduced with CoQ10 or *n*-3 PUFAs supplementation. At P21, the levels rebounded in the vitreous fluid of the controls exposed to IH and in the CoQ10 supplemented group exposed to 21/12% O_2_ IH. Levels in the retina and choroid remained suppressed particularly with *n*-3 PUFA supplementation. These findings also suggest that *n*-3 PUFAs are more potent suppressors of sVEGFR-1.

### 3.4. Ocular IGF-I

IGF-I is a permissive factor for VEGF, and low systemic levels have been shown to be a predictor for severe ROP in preterm infants. Levels of IGF-I in the vitreous fluid, retina, and choroid are presented in Table 3. At P14, only *n*-3 PUFAs suppressed IGF-I levels in the vitreous fluid of animals exposed to 50/12% O_2_ IH. However, in the retina and choroid, *n*-3 PUFAs supplementation increased IGF-I levels in RA and IH. Similar elevations were noted with CoQ10 supplementation in IH. At P21, CoQ10 decreased IGF-I levels in the retinas exposed to RA, while elevations remained sustained with *n*-3 PUFAs in RA. Treatment with CoQ10 or *n*-3 PUFAs increased retinal and choroidal IGF-I levels in IH. These data suggest that supplementation with CoQ10 or *n*-3 PUFAs cause elevations in IGF-I levels. 

### 3.5. Retinal Angiogenesis 

**Figure 4:**
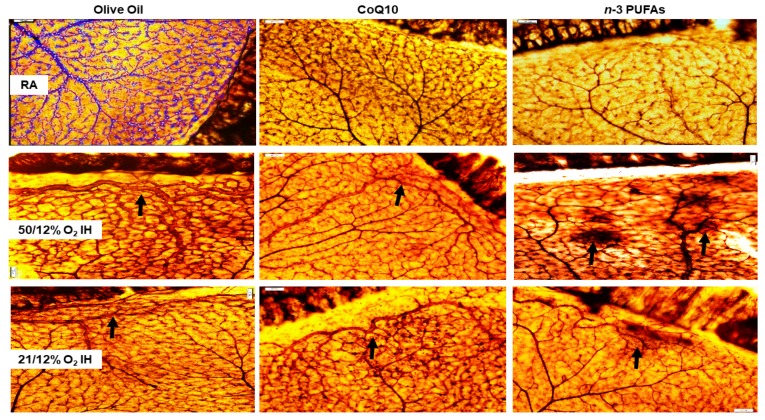
Representative adenosine diphosphatase (ADPase)-stained retinal flatmount from 21-day-old rat exposed to RA (**top panels**), 50/12% O_2_ IH (**middle panels**), and 21/12% O_2_ IH (**lower panels**). Animals were supplemented with olive oil (**left panels**), CoQ10 (**middle panels**), or *n*-3 PUFAs (**right panels**). The representative flatmount from the RA-exposed, olive oil-treated rats shows example of WimRetina analysis for the quantitation of the vascular density parameters listed in Table 5. Images are 10× magnification. Scale bar, 100 µm.

ADPase-stained images of the retinas at P21 are presented in Figure 4. The olive oil RA group is shown as a representation of the WimRetina analysis that was used for the quantitation of vascular density parameters. At P21, the RA-exposed retinas appear normal, with no evidence of any of the characteristics consistent with severe OIR, although moderate capillary dropout was seen in the *n*-3 PUFA group. In 50/12% O_2_ IH, the retinas from animals in the olive oil group showed dilated abundant vascular networks, disorganized tortuous vessels, neovascularization, and anastomoses at the periphery (arrow). CoQ10 treatment reduced vessel size, capillary plexus, and vascular network, but anastomoses persisted. Treatment with *n*-3 PUFAs also reduced vessel size, capillary plexus, and vascular network; however, there was evidence of intraretinal hemorrhages. In 21/12% O_2_ IH, the retinal vascular abnormalities appeared mitigated, although evidence of abundant vascular networks remained in the olive oil and CoQ10 groups. Treatment with *n*-3 PUFAs decreased branching elements and capillary plexus, but intraretinal hemorrhages persisted, although these were less abundant (arrow).

### 3.6. Retinal Scoring

Retinal scoring for the severity of OIR revealed that none of the RA groups had a score of >3 for any of the characteristics. The average total score in RA for olive oil, CoQ10, and *n*-3 PUFAs was nine, seven and eight, respectively. In contrast, animals exposed to IH and treated with olive oil showed 75% and received a score >3 for intraretinal hemorrhages, 60% for arterial tortuosity, 75% for vascular tufts, 50% for vascular overgrowth, and 25% for persistence of hyaloid vessels (all *p* < 0.01 compared to RA). CoQ10 and *n*-3 PUFAs reduced the percentage of retinas that scored >3 for intraretinal hemorrhages to 25% (*p* < 0.01), tortuosity to 40% (*p* < 0.01), vascular tufts to 0 (*p* < 0.01), vascular overgrowth to 25% (*p* < 0.01), and persistence of hyaloid vessels to 0 (*p* < 0.01) in both IH paradigms. 

### 3.7. Astrocytic Template 

**Figure 5:**
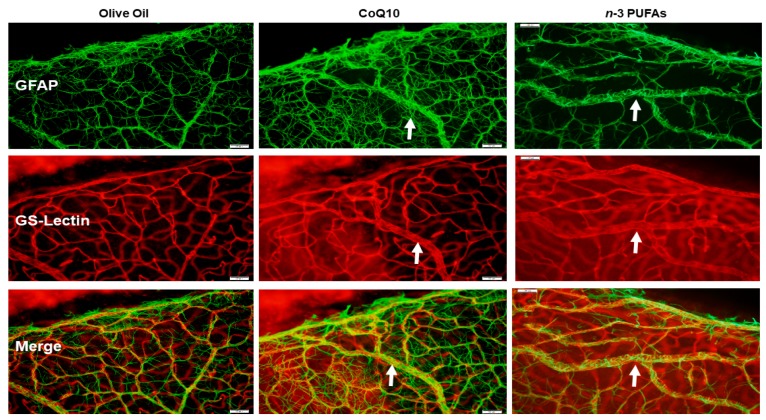
Representative images of retinal flatmounts from 21-day-old rat exposed to RA. Astrocytes and Müller cells were stained for glial fibrillary acidic protein (GFAP) immunoreactivity (green), and retinal vasculature was stained with isolectin B4, which is a biomarker for endothelial cells (red). Merged images are presented in the bottom panel. The white arrows in the CoQ10 and *n*-3 PUFA groups show mild vessel dilation at the periphery. Animals were supplemented with olive oil (**left panels**), CoQ10 (**middle panels**), or *n*-3 PUFAs (**right panels**). Images are 10× magnification, and the scale bar is 100 µM.

**Figure 6:**
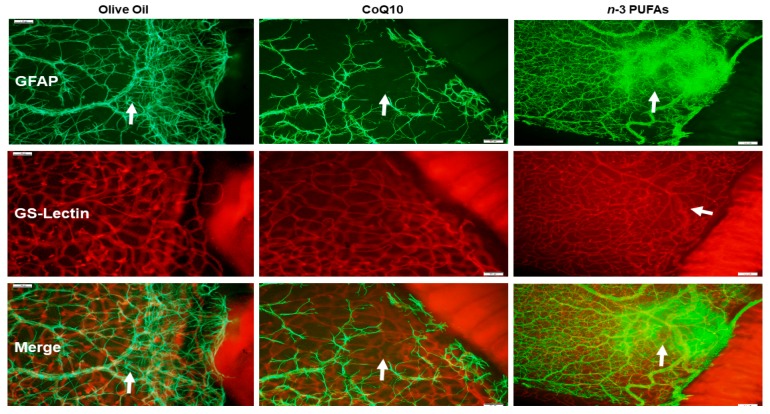
Representative images of retinal flatmounts from 21-day-old rat exposed to 50/12% O_2_ IH. Astrocytes and Müller cells were stained for GFAP immunoreactivity (green), and retinal vasculature was stained with isolectin B4, which is a biomarker for endothelial cells (red). Merged images are presented in the bottom panel. Animals were supplemented with olive oil (**left panels**), CoQ10 (**middle panels**), or *n*-3 PUFAs (**right panels**). Images are 10× magnification and the scale bar is 100 µM.

**Figure 7:**
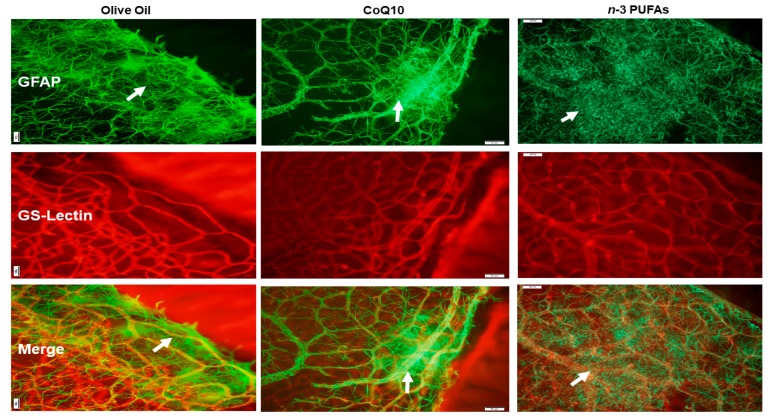
Representative images of retinal flatmounts from 21-day-old rat exposed to 21/12% O_2_ IH. Astrocytes and Müller cells were stained for GFAP immunoreactivity (green), and retinal vasculature was stained with isolectin B4, which is a biomarker for endothelial cells (red). Merged images are presented in the bottom panel. Animals were supplemented with olive oil (**left panels**), CoQ10 (**middle panels**), or *n*-3 PUFAs (**right panels**). Images are 10× magnification, and the scale bar is 100 µM.

Astrocytes are found only in the NFL/GCL and are GFAP positive. They exist proximal to the inner retinal vasculature, providing guidance cues and structure. Isolectin B4 is a specific endothelial cell marker that labels blood vessels. GFAP and isolectin B4-stained retinas at P21 with supplementation in RA are shown in Figure 5. The upper panels represent the GFAP-stained retinas, the middle panels represent the isolectin B4-stained retinas, and the lower panels are the merged images. The control group supplemented with olive oil showed no major abnormalities. However, treatment with CoQ10 or *n*-3 PUFAs in RA resulted in some retinas with dilated vessels at the periphery (arrows). In 50/12% O_2_ IH (Figure 6), animals treated with olive oil showed astrocyte disorganization, CoQ10 reduced GFAP staining, and *n*-3 PUFAs caused GFAP hypertrophy, and activated Müller end-feet (arrow). In 21/12% O_2_ IH (Figure 7), astrocyte disorganization and hypertrophy worsened in all of the groups, but were most severe in the *n*-3 PUFAs group showing major disruptions in the astrocytic template (arrows).

### 3.8. Retinal Vascular Quantification

WimRetina quantification of the retinal vasculature at P21 is presented in Table 4. Data revealed that vascular density was increased in both IH groups, but total vascular area was highest in the 50/12% O_2_ IH group. In RA, exposure to *n*-3 PUFAs reduced the total vascular area, number of branching points, and number of segments, but increased segment length. CoQ10 and *n*-3 PUFAs decreased vascular density, total vascular area, number of branching points, and number of segments in both IH paradigms, but increased segment length in 50/12% O_2_ IH.

### 3.9. Retinal Histopathology

**Figure 8:**
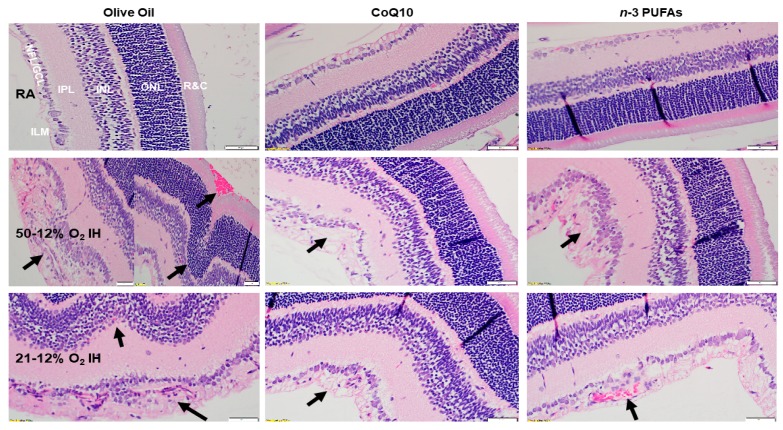
Representative Hematoxylin & eosin (H&E) stained images of retinas from 21-day-old rat exposed to RA (**top panels**), 50/12% O_2_ IH (**middle panels**), and 21/12% O_2_ IH (**lower panels**). Animals were supplemented with olive oil (**left panels**), CoQ10 (**middle panels**), or *n*-3 PUFAs (**right panels**). The images show retinal layer integrity and number of endothelial cells in the nerve fiber layer (NFL)/ganglion cell layer (GCL). The layers are identified in the RA-exposed, olive oil-treated animal. ILM (inner limiting membrane); IPL (inner plexiform layer); INL (inner nuclear layer); ONL (outer nuclear layer); and R&C (rods and cones). Images are 40× magnification, and the scale bar is 50 µM.

Hematoxylin & eosin (H&E) stained retinal sections from rats at P21 are presented in Figure 8. The room air control groups showed no evidence of retinal pathology. However, the group supplemented with olive oil and exposed to IH showed retinal endothelial cells breaching the inner limiting membrane (ILM) and violating the vitreous fluid (arrow), as well as vitreous condensation and widening of the NFL/GCL. The outer retina showed major distortions in the ONL with the appearance of retinal folds or rosettes (arrow), hemorrhage (arrow), and possibly retinal detachment. These characteristics are consistent with apoptosis, degeneration, and cell death. Supplementation with CoQ10 and *n*-3 PUFAs reduced the number of cells in the NFL/GCL layer and curtailed migration into the vitreous fluid, but did not completely prevent it (arrow).

### 3.10. Retinal Morphometry

Retinal morphometry at P21 is presented in Table 5. The tortuosity index and vessel diameter were quantitated using ADPase-stained retinas, and H&E stained retinas were used for the quantification of retinal thickness. In RA, *n*-3 PUFAs decreased vessel size. However, CoQ10 or *n*-3 PUFAs increased the number of cells in the NFF/GCL layer. CoQ10 decreased overall retinal thickness as well as INL and ONL thickness, while *n*-3 PUFAs reduced IPL thickness. Exposure to 50/12% O_2_ IH increased all of the parameters in the olive oil group, which was an effect that was significantly reduced with CoQ10 or *n*-3 PUFAs supplementation. Exposure to 21/12% O_2_ IH resulted in elevated tortuosity index, diameter of veins, number of cells in the NFL/GCL, overall retinal thickness, and NFL/GCL thickness. CoQ10 decreased the tortuosity index, diameter of vessels, number of cells in the NFL/GCL, and ONL thickness compared to control, while supplementation with *n*-3 PUFAs decreased the number of cells in NFL/GCL, and NFL/GCL thickness.

## 4. Discussion

The present study employed two clinically-relevant paradigms to test the hypothesis that supplementation with either CoQ10 or *n*-3 PUFAs during neonatal IH can reduce the severity of OIR. This hypothesis was based on previous reports of reduction in the inflammation and severity of major neonatal disease with *n*-3 PUFAs supplementation [28,29,30], and the bioenergetics properties of CoQ10 [31,32,33,34,35,36,37,38,39]. The two IH paradigms that were used in these studies address an important clinical question regarding whether bagging the baby with room air to resolve an IH episode is more beneficial to the immature eye than resolving an IH event with oxygen. The data showed that both IH paradigms are injurious to the developing retina, producing characteristics that are consistent with severe ROP, although exposure to 50/12% O_2_ IH worsened the outcomes. These data confirm that the immature retina is highly vulnerable to any variations in oxygen, and efforts to curtail these variations should remain a high priority. 

The first evidence of the beneficial effects of CoQ10 or *n*-3 PUFAs supplementation during neonatal IH is demonstrated in Table 1. *n*-3 PUFAs accelerated visual maturation and shortened the caecal period in over 80% of treated rats in RA and 50/12% O_2_ IH, and to a lesser degree in 21/12% O_2_ IH. Eye-opening in rodents is a key indicator of retinal maturation and development of the retinal neural circuitry [49,50]. These findings support previous reports of improved visual acuity and maturation in infants supplemented with DHA [51,52]. The importance of *n*-3 PUFAs for retinal health is reported in a large body of work. In particular, DHA influences both neuronal and retinal vascular cell survival and development [53]. Further, studies show that fish oil fat emulsion supplementation reduces the risk of ROP in preterm infants [54,55]. The influence of CoQ10 on retinal maturation and retinal neural circuitry is less clear. However, previous studies have reported the benefits of CoQ10 supplementation as a retinal neuroprotective agent against oxidative damage [56,57,58]. In our studies, we found that CoQ10 was not as effective as *n*-3 PUFAs for improving retinal maturation and neural circuitry development, particularly in the 50/12% O_2_ IH paradigm. The protective effect of DHA on visual function has been previously demonstrated [59], and our findings provide further support. The outer retina is rich in PUFAs, levels of CoQ10 are relatively low: about 42 nmol/gram dry retina [60]. These low levels coupled with the retina’s high metabolic rate and the need for an efficient antioxidant suggest that CoQ10 is a powerful antioxidant in the eye, and functions to protect membrane phospholipids from lipid peroxidation and oxidative damage [61]. Taken together, these data imply that in the setting of IH, *n*-3 PUFAs supplementation is more beneficial than CoQ10 for retinal neural maturation. Further studies are needed to determine whether increasing the doses of CoQ10 will provide similar beneficial outcomes.

The second test of our hypothesis is demonstrated in Figure 1, Figure 2 and Figure 3, which describe the effects of CoQ10 or *n*-3 PUFAs supplementation during IH on ocular biomarkers of angiogenesis. Vascular growth factors are critical for ocular angiogenesis, and VEGF is the most potent angiogenesis-promoting factor. It is highly induced by hypoxia, and acts via activation of its receptors expressed on vascular endothelial cells. The role of VEGF in the development of ROP is well described in numerous reports. ROP is generally characterized by two phases [8,62,63,64,65]. The first is an early vaso-obliterative phase (Phase 1) leading to ocular hypoxia and aberrant regulation of ocular vascular growth factors. During this phase, exposure to supplemental oxygen suppresses retinal growth factors such as VEGF and IGF-I, which are already compromised due to preterm birth and poor nutrition. This leads to arrest and retraction of the developing retinal vessels. This is followed by a vaso-proliferative phase (Phase 2), which begins at approximately 32–34 weeks [66]. As the infant matures, the avascular retina becomes metabolically active, inducing retinal neovascularization. This phase of ROP is driven by hypoxia and the subsequent upregulation of VEGF and IGF-I, which leads to abnormal vascular overgrowth into the vitreous, retinal hemorrhages, retinal folds, dilated and tortuous posterior retinal blood vessels, or “Plus” disease, and retinal detachment [67]. Thus, it was important to determine the levels of ocular growth factor immediately post IH exposure. Interestingly, VEGF was highly upregulated at P14 in all of the ocular compartments. This confirms that in our model of neonatal IH, the two phases of ROP are occurring almost simultaneously so that grouping IH events (simulating arterial oxygen desaturations in preterm infants at risk for severe ROP), with minimal time for recovery between episodes causes the retina to remain hypoxic for longer periods of time. While CoQ10 or *n*-3 PUFAs did not appreciably reduce VEGF levels in the vitreous fluid following the 50/12% O_2_ IH paradigm, they were effective in the 21/12% O_2_ IH paradigm, and were both effective for reducing retinal VEGF levels. Only CoQ10 reduced choroidal VEGF levels in both IH paradigms. Surprisingly, *n*-3 PUFAs supplementation resulted in a predominance of intraretinal hemorrhages at the periphery. While we have no explanation for this finding, it should be noted that *n-*3 PUFAs have been reported to have anti-thrombotic effects [68]. A high *n-*3 PUFAs diet was associated with hemorrhagic stroke [69]. Therefore, it is likely, though not proven, that the predominance of retinal hemorrhages seen in our study with *n*-3 PUFA supplementation may be due to its anti-thrombotic effects. 

We previously reported that the vitreous acts as a reservoir for growth factors in the eyes, and levels are correlated with serum levels [70]. Others suggest that vitreous fluid VEGF levels are a predictor for ocular diseases [71]. In our study, high levels of VEGF in the vitreous fluid could be due to intraretinal hemorrhage and/or disruption of the blood–retina barrier (BRB), which occurs in IH and retinopathy. Intraretinal hemorrhage is one of the critical characteristics that is often seen in our IH model (shown in Figure 4), and used for retinal scoring to determine the severity of OIR. Hemorrhage and BRB breakdown can produce large amounts of serum proteins in the vitreous. However, although no samples were contaminated with blood in this study, and all of the vitreous fluid samples were centrifuged prior to analyses, we cannot rule out the influence of IH-induced hemorrhage and BRB breakdown. These data imply that although VEGF was successfully reduced with CoQ10 and *n*-3 PUFAs supplementation, high levels in the vitreous acting as a reservoir may be extracted by the retina during the recovery phase (P21) in RA. As noted in Figure 8, cells violating the vitreous fluid and possible disruption of the inner limiting membrane (INL), albeit to a lesser degree, were evident at P21 despite treatment. This may explain, at least in part, the failure to completely prevent severe OIR, and suggest that a longer term supplementation may prove to be more beneficial. Nevertheless, early supplementation with CoQ10 or *n*-3 PUFAs during IH may provide moderate protection against IH-induced retinal vascular damage by reducing VEGF levels. 

Soluble VEGFR-1 (sVEGFR-1) is an endogenous negative regulator of VEGF. It was of great interest that supplementation with CoQ10 or *n*-3 PUFAs suppressed sVEGFR-1 in both IH paradigms. sVEGFR-l expression is up-regulated by VEGF in a comparable fashion [72], possibly to regulate itself. Given the role of sVEGFR-1 as a VEGF “trap” by binding VEGF and preventing its availability to the membrane VEGF receptors [73], this decline in sVEGFR-1 is likely reflective of the decline in VEGF. The importance of sVEGFR-1 in the eye is demonstrated by the high levels in the photoreceptor layer, which lies between the vascular inner retina and the highly vascularized choroid (which has the highest flow of any vascular bed), fulfilling the metabolic and oxygen demands of the outer retina. sVEGFR-1 is synthesized by photoreceptors and retinal pigment epithelium (RPE), where it acts to protects the avascular photoreceptor layer from vascular invasion [74]. Although reduction in choroidal sVEGFR-1 could lead to choroidal vessels breaching the RPE layer, concurrent reductions in VEGF prevents the VEGF/sVEGFR-1 imbalance, favoring VEGF. IGF-1 serves as a permissive factor for VEGF action. Low systemic levels have been shown to be a strong predictor of severe ROP in preterm infants [75]. The data showed that *n*-3 PUFAs supplementation is a more potent inducer of IGF-I. This effect of *n*-3 PUFA supplementation on IGF-I has been previously shown [76]. While the mechanism remains unclear, it should be noted that both IGF-I and *n*-3 PUFAs are depleted in preterm infants due to the loss of transfer in the third trimester from mother to infant [77]. Therefore, early supplementation with *n*-3 PUFAs may have a dual beneficial effect.

The third test of our hypothesis is demonstrated by Figure 5, Figure 6 and Figure 7, which compare the effects of CoQ10 and *n*-3 PUFAs on the retinal astrocyte integrity. The role of retinal astrocytes during development of the superficial retinal vasculature is well-described [78,79,80]. In normal retinal development, retinal astrocytes emerge from the optic nerve head preceding the retinal vasculature and form a scaffold or template. They produce VEGF to promote retinal endothelial cell (EC) proliferation, migration, and vascular patterning. Therefore, any disturbances in the astrocytic template strongly influence VEGF production and normal vascular patterning. Astrocytes are confined to the NFL/GCL layer, and are intimately associated with the retinal blood vessels [81]. They ensheathe the vessels, providing stability and protection. This restriction to the inner retina enables the astrocytes to rapidly respond to hypoxic injury with a resulting increased expression of VEGF and vessel formation. However, in that process, they become highly GFAP-reactive, disorganized, and lose their stellate shape [82]. Activated astrocytes have been found to be related to retinal neuronal injury [83] and increased vascular permeability [84]. In the present study, CoQ10 appeared to be more beneficial for preventing astrocyte reactivity in 50/12% O_2_ IH, and to a lesser degree, in 21/12% O_2_ IH. On the contrary, *n*-3 PUFAs was not effective. These findings establish a possible novel role for CoQ10 in protection against IH-induced gliosis, support that astrogliosis is highly induced by oxidative distress, and demonstrate the antioxidant capacity of CoQ10 in the eye. 

The final test of our hypothesis is demonstrated by Table 4 and Table 5, and supported by Figure 4 and Figure 8, which compares the effects of CoQ10 and *n*-3 PUFAs on retinal neovascularization quantitation and retinal morphometry. One of the major abnormalities noted in our neonatal IH models is the rearrangement of the photoreceptor cells into folds and rosettes, particularly at P21 during the reoxygenation phase. This same phenomenon has been shown in preterm infants and in animal models [85,86], and is indicative of apoptosis, degeneration, partial retinal detachment, and loss of RPE and/or function. As shown in Figure 8, exposure to 50/12% O_2_ IH not only resulted in severe NFL/GCL disruption, but folding of the outer retina was concurrent with choroidal hemorrhage and possible retinal detachment, all of which were curtailed with CoQ10 and *n*-3 PUFAs supplementation. Retinal thickness, as determined by optical coherence tomography, has been previously shown to predict “plus” disease or stage 3 ROP in preterm infants [87]. In our IH models, we noted increased retinal thickness and vascular tortuosity (a characteristic consistent with “plus” disease) in the olive oil supplemented groups exposed to IH. Thickness of the whole retina was predominantly due to expansion of the NFL/GCL and ONL layers. It is likely that Müller cells, the principal glial cells of the retina and major producer of VEGF, participate in the IH-induced increased retinal thickness. Müller cell swelling and gliosis, evidenced by GFAP activation, is a known indicator of retinal stress and edema via up-regulations of aquaporin 4 and the down-regulation of aquaporin 1 in the ONL, leading to ONL abnormalities, breakdown of the BRB, and photoreceptor cell death [88]. CoQ10 and *n*-3 PUFAs were effective in reversing these IH-induced aberrations. Given the role of CoQ10 as an electron-shuttling compound in OXPHOS, one limitation of this study is that we did not determine mitochondrial potential/function or oxidative stress in the ocular compartment. Levels of antioxidants in the systemic circulation in response to CoQ10 or *n*-3 PUFAs supplementation during neonatal IH have been previously reported [40]. 

## 5. Conclusions

The multifactorial pathogenesis of ROP may preclude the use of a single agent to target the complex interactions of growth factors; vascular, astrocytic, and membrane disruption; oxidative distress; and inflammatory responses. Individually, CoQ10 and *n*-3 PUFAs supplementation in IH have protective effects on characteristics that are consistent with severe ROP, but they did not completely prevent severe OIR. CoQ10 reduces VEGF, preserves astrocytic integrity, reduces neovascularization, and normalizes retinal layers, particularly in 50/12% O_2_ IH. Whereas, *n*-3 PUFAs decrease VEGF, promote IGF-I secretion, reduce neovascularization, and normalize retinal layers. As an alternate therapeutic approach, it would be interesting to see if combined CoQ10 and *n*-3 PUFAs supplementation would optimize their efficacy and result in a better outcome. Further studies are warranted.

## Figures and Tables

**Table 1:** Eye-Opening at Postnatal Day 14 (P14).

Eyes	RA			50%/12% O_2_			21%/12% O_2_		
	Olive Oil	CoQ10	*n*-3 PUFAs	Olive Oil	CoQ10	*n*-3 PUFAs	Olive Oil	CoQ10	*n*-3 PUFAs
**Left Eye**	15 (42%)	11 (31%)	32 (89%) ***	11 (31%)	19 (53%)	34 (94%) ^###^	19 (53%)	28 (78%) ^‡^	29 (81%) ^§^
**Right Eye**	14 (39%)	12 (33%)	32 (89%) ***	9 (25%)	19 (53%)	35 (97%) ^###^	22 (61%)	32 (89%) ^‡^	28 (78%)
**Both Eyes**	11 (31%)	10 (28%)	31 (86%) ***	7 (19%)	18 (50%)	33 (62%) ^###^	18 (50%)	27 (75%) ^‡^	25 (69%)

CoQ10: coenzyme Q10, PUFA: polyunsaturated fatty acids, RA: room air. All of the animals were examined at P14 (*n* = 36 rats/group; *** *p* < 0.001 vs. olive oil RA; **^###^***p* < 0.001 vs. Olive Oil 50/12% O_2_; ^‡^ *p* < 0.001 vs. CoQ10 RA; and ^§^ *p* < 0.05 vs. olive oil 21/12% O_2_).

**Table 2:** Ocular sVEGFR-1.

Ocular Compartment	Olive Oil	CoQ10	*n*-3 PUFAs
***P14-RA:***			
Vitreous (pg/mL):	195.9 ± 61.0	127.5 ± 60.3	64.2 ± 17.6
Retina (pg/mg protein):	96.0 ± 12.5	181.6 ± 96.8 *	65.8 ± 11.5
Choroid: (pg/mg protein):	419.0 ± 102.4	297.1 ± 133.0	913.7 ± 128.8 **
***P14-50/12% IH:***			
Vitreous (pg/mL):	330.9 ± 59.3 ^§^	93.2 ± 50.9 ^##^	42.7 ± 11.5 ^##^
Retina (pg/mg protein):	240.4 ± 26.0 **	54.2 ± 13.2 ^##§§^	87.4 ± 16.7 ^##§§^
Choroid: (pg/mg protein):	1329.9 ± 198.9 **	681.3 ± 329.2 ^##§^	1044.4 ± 196.2
***P14-21/12% IH:***			
Vitreous (pg/mL):	161.5 ± 95.6	50.0 ± 36.0 ^§^	37.9 ± 35.0 ^‡§§^
Retina (pg/mg protein):	237.5 ± 11.3 **	49.9 ± 9.1 ^‡§§^	51.8 ± 11.5 ^‡^
Choroid: (pg/mg protein):	1191.3 ± 292.7 **	525.2 ± 189.1 ^‡^	392.9 ± 124.7 ^‡§^
***P21-RA:***			
Vitreous (pg/mL):	183.5 ± 20.3	216.8 ± 116.6	165.2 ± 27.2
Retina (pg/mg protein):	85.9 ± 5.1	104.1 ± 33.3	116.1 ± 2.9 *
Choroid: (pg/mg protein):	1217.8 ± 162.4	632.3 ± 234.4 **	997.3 ± 131.3 **
***P21-50/12% IH:***			
Vitreous (pg/mL):	260.9 ± 34.3 ^§§^	225.4 ± 79.4	198.8 ± 60.0
Retina (pg/mg protein):	112.4 ± 55.4	89.5 ± 34.5	80.8 ± 9.8 ^§§^
Choroid: (pg/mg protein):	1580.0 ± 223.4 **	514.8 ± 111.0 ^##^	757.7 ± 55.4 ^##§§^
***P21-21/12% IH:***			
Vitreous (pg/mL):	245.9 ± 29.4 ^§§^	287.6 ± 132.8 ^§§^	273.3 ± 55.1
Retina (pg/mg protein):	40.6 ± 23.8	94.1 ± 6.4 ^‡^	53.2 ± 9.6 ^§§^
Choroid: (pg/mg protein):	595.0 ± 95.5 **	1215.4 ± 176.4 ^‡§§^	624.6 ± 130.1 ^§§^

Data are mean ± SD. *n* = six samples/group (* *p* < 0.05, ** *p* < 0.01 vs. olive oil in RA; ^##^ *p* < 0.01 vs. olive oil in 50/12% O_2_ IH; ^‡^ *p* < 0.01 vs. olive oil in 21/12% O_2_; and ^§^ *p* < 0.05; ^§§^ *p* < 0.01 vs. RA). RA (room air); IH (intermittent hypoxia); VEGF (vascular endothelial growth factor); sVEGFR-1 (soluble vascular endothelial growth factor receptor-1); IGF-I (insulin-like growth factor-I).

**Table 3:** Ocular IGF-I.

Ocular Compartment	Olive Oil	CoQ10	*n*-3 PUFAs
***P14-RA:***			
Vitreous (ng/mL):	795.1 ± 34.0	753.5 ± 130.1	793.1 ± 72.3
Retina (ng/mg protein):	8.4 ± 3.9	16.5 ± 11.8	23.6 ± 5.4 **
Choroid: (ng/mg protein):	30.5 ± 14.9	34.4 ± 33.8	173.3 ± 53.2 **
***P14-50/12% IH:***			
Vitreous (ng/mL):	924.3 ± 127.1	872.2 ± 64.4	767.5 ± 62.7 ^#^
Retina (ng/mg protein):	27.2 ± 13.5 **	12.9 ± 10.8	21.1 ± 11.8
Choroid: (ng/mg protein):	12.1 ± 20.8	105.2 ± 23.5 ^#§§^	204.4 ± 86.5 ^##^
***P14-21/12% IH:***			
Vitreous (ng/mL):	763.0 ± 276.8	803.6 ± 85.0	831.5 ± 54.1
Retina (ng/mg protein):	2.2 ± 2.9	25.2 ± 8.1 ^‡^	29.5 ± 4.7 ^‡^
Choroid: (ng/mg protein):	94.3 ± 52.2 **	125.4 ± 34.5 ^§§^	49.4 ± 17.9 ^§§^
***P21-RA:***			
Vitreous (ng/mL):	708.7 ± 39.9	690.0 ± 108.0	509.7 ± 17.6
Retina (ng/mg protein):	25.8 ± 9.6	5.9 ± 4.2 **	40.0 ± 2.9 **
Choroid: (ng/mg protein):	83.4 ± 8.6	33.1 ± 9.6 **	80.6 ± 41.2
***P21-50/12% IH:***			
Vitreous (ng/mL):	647.0 ± 67.1	756.2 ± 64.4	594.6 ± 45.8
Retina (ng/mg protein):	13.9 ± 8.3 *	26.0 ± 10.8 ^#§§^	13.1 ± 5.9 ^§§^
Choroid: (ng/mg protein):	0 ± 0 **	49.2 ± 8.1 ^##§^	81.7 ± 23.0 ^##^
***P21-21/12% IH:***			
Vitreous (ng/mL):	603.1 ± 56.6	711.4 ± 89.9	888.1 ± 24.3
Retina (ng/mg protein):	1.3 ± 1.5 **	21.6 ± 6.1 ^‡§§^	14.0 ± 2.7 ^‡§§^
Choroid: (ng/mg protein):	46.7 ± 17.1 **	57.4 ± 10.3 ^§§^	36.5 ± 10.8 ^§^

Data are mean ± SD. *n* = six samples/group (* *p* < 0.05; ** *p* < 0.01 vs. olive oil in RA; **^#^***p* < 0.05; ^##^ *p* < 0.01 vs. olive oil in 50/12% O_2_ IH; ^‡^ *p* < 0.01 vs. olive oil in 21/12% O_2_; and ^§^ *p* < 0.05, ^§§^ *p* < 0.01 vs. RA). RA (room air); IH (intermittent hypoxia); VEGF (vascular endothelial growth factor); sVEGFR-1 (soluble vascular endothelial growth factor receptor-1); IGF-I (insulin-like growth factor-I).

**Table 4:** WimRetina Quantitation of Retinal Vasculature at P21.

Vascular Parameters	Olive Oil	CoQ10	*n*-3 PUFAs
***RA:***			
Vascular Density (%)	34.9 ± 3.33	36.8 ± 7.3	33.7 ± 4.8
Total Vascular Area	28,068.8 ± 3366.1	27,084.5 ± 4843.6	22,814.7 ± 3429.8 **
No. Branching Points	568.9 ± 151.9	567.0 ± 245.4	424.9 ± 129.3 *
No. Segments	1084.7 ± 205.3	1042.4 ± 392.9	813.4 ± 224.4 **
Mean Segment Length	25.7 ± 3.6	27.0 ± 4.4	28.9 ± 3.7 *
***50/12% IH:***			
Vascular Density (%)	46.4 ± 16.7 **	32.9 ± 5.9 ^##^	32.2 ± 2.6 ^##^
Total Vascular Area	38,444.3 ± 10,394.0 **	25,052.0 ± 5403.6 ^##^	21,725.2 ± 1943.4 ^##^
No. Branching Points	839.9 ± 668.7	483.1 ± 230.7 ^#^	328.3 ± 51.4 ^##§§^
No. Segments	1458.1 ± 1035.2	905.6 ± 382.6 ^#^	655.5 ± 345.9 ^##^
Mean Segment Length	23.6 ± 7.3	29.3 ± 4.8 ^##^	33.3 ± 2.4 ^##§§^
***21/12% IH:***			
Vascular Density (%)	38.7 ± 3.6 **	31.9 ± 2.9 ^‡§^	33.3 ± 3.1 ^‡^
Total Vascular Area	29,057.7 ± 2680.2	24,396.9 ± 2427.0 ^‡^	26,001.0 ± 3100.1 ^‡§§^
No. Branching Points	652.4 ± 108.8	444.3 ± 94.1 ^‡^	500.5 ± 94.6 ^‡§^
No. Segments	1110.4 ± 182.3	848.1 ± 158.2 ^‡^	933.2 ± 161.2 ^‡^
Mean Segment Length	28.4 ± 2.5	29.5 ± 2.6	28.0 ± 2.1

Data are mean ± SD. *n* = 24 samples/group (* *p* < 0.05; ** *p* < 0.01 vs. olive oil in RA; ^#^ *p* < 0.05; ^##^ *p* < 0.01 vs. olive oil in 50/12% O_2_ IH; ^‡^ *p* < 0.01 vs. olive oil in 21/12% O_2_; and ^§^ *p* < 0.05; ^§§^ *p* < 0.01 vs. RA). RA (room air); IH (intermittent hypoxia).

**Table 5:** Retinal Morphometry at P21.

Parameters	Olive Oil	CoQ10	*n*-3 PUFAs
***RA:***			
Tortuosity Index	0.96 ± 0.05	0.97 ± 0.05	0.98 ± 0.03
Diameter of Arteries	55.3 ± 9.3	50.6 ± 11.8	46.1 ± 7.3 **
Diameter of Veins	41.9 ± 10.3	38.4 ± 9.8	32.2 ± 5.9 **
Number of Cells in NFL/GCL	106.0 ± 19.6	115.0 ± 1.8 *	125.6 ± 1.3 **
Retinal Thickness (µm)	342.2 ± 65.6	291.6 ± 76.9 *	356.9 ± 71.5
NFL/GCL Thickness (µm)	48.0 ± 20.1	41.6 ± 21.1	48.3 ± 11.8
IPL Thickness (µm)	65.5 ± 21.1	61.0 ± 17.6	40.0 ± 16.2 **
INL Thickness (µm)	74.3 ± 13.7	58.9 ± 13.7 **	69.5 ± 12.7
ONL Thickness (µm)	109.6 ± 36.7	78.7 ± 12.7 **	103.3 ± 21.1
***50/12% IH:***			
Tortuosity Index	1.52 ± 0.24 **	1.1 ± 0.1 ^##§§^	1.08 ± 0.04 ^##§§^
Diameter of Arteries	60.9 ± 3.4 *	45.1 ± 12.2 ^##^	42.7 ± 10.3 ^##^
Diameter of Veins	48.3 ± 8.3 *	37.6 ± 5.9 ^##^	34.9 ± 7.3 ^##^
Number of Cells in NFL/GCL	231.8 ± 12.7 **	133.9 ± 1.8 ^##§§^	142.2 ± 2.0 ^##§§^
Retinal Thickness (µm)	433.9 ± 97.5 **	280.1 ± 45.1 ^##^	408.1 ± 117.6
NFL/GCL Thickness (µm)	103.4 ± 44.6 **	44.5 ± 22.0 ^##^	55.9 ± 28.4 ^##^
IPL Thickness (µm)	86.4 ± 16.2 **	52.2 ± 8.8 ^##^	71.6 ± 21.6 ^§§^
INL Thickness (µm)	105.4 ± 44.6 **	60.6 ± 13.7 ^##^	83.1 ± 25.5 ^#^
ONL Thickness (µm)	175.0 ± 91.1 **	82.3 ± 12.2 ^##^	129.0 ± 73.0 ^#^
***21/12% IH:***			
Tortuosity Index	1.21 ± 0.15 **	1.09 ± 1.1 ^‡§§^	1.17 ± 0.15 ^§§^
Diameter of Arteries	50.7 ± 8.3	44.6 ± 8.3 ^†^	47.9 ± 8.3
Diameter of Veins	35.8 ± 6.4 *	31.7 ± 5.9 ^†§§^	34.8 ± 6.4
Number of Cells in NFL/GCL	148.8 ± 2.9 **	139.9 ± 1.4 ^‡§§^	131.7 ± 1.7 ^‡§§^
Retinal Thickness (µm)	424.8 ± 82.3 **	411.8 ± 65.2 ^§§^	392.9 ± 72.0
NFL/GCL Thickness (µm)	78.6 ± 23.5 **	65.7 ± 30.9 ^§§^	59.3 ± 23.0 ^†^
IPL Thickness (µm)	63.7 ± 12.2	69.2 ± 18.1	66.0 ± 14.2 ^§§^
INL Thickness (µm)	85.8 ± 12.2	78.1 ± 31.8 ^§§^	78.3 ± 27.9
ONL Thickness (µm)	148.6 ± 44.6	110.9 ± 29.4 ^†§§^	132.6 ± 79.4

Data are mean ± SD (* *p* < 0.05, ** *p* < 0.01 vs. olive oil in RA; ^#^ *p* < 0.05; ^##^ *p* < 0.01 vs. olive oil in 50/12% O_2_ IH; ^†^ *p* < 0.05 vs. olive oil in 21/12% O_2_; and ^§§^ *p* < 0.01 vs. RA). *n* = 24 measurements/group; RA (room air); IH (intermittent hypoxia); NFL/GCL (nerve fiber layer/ganglion cell layer); IPL (inner plexiform layer); INL (inner nuclear layer); ONL (outer nuclear layer).
